# Dispersive magnetic solid-phase extraction for capsaicinoid compounds in human serum using LC-HRMS: targeted and non-targeted approaches

**DOI:** 10.1007/s00216-023-04544-7

**Published:** 2023-01-25

**Authors:** María Consolación Rodríguez-Palazón, Natalia Arroyo-Manzanares, Pilar Viñas, Ignacio López-García, Manuel Hernández-Córdoba, Natalia Campillo

**Affiliations:** Department of Analytical Chemistry, Faculty of Chemistry, Regional Campus of International Excellence “Campus Mare Nostrum”, University of Murcia, E-30100 Murcia, Spain

**Keywords:** Capsaicinoids, Serum, Dispersive magnetic solid-phase extraction, Ultra-high-performance liquid chromatography, Quadrupole-time-of-flight mass spectrometry, Non-targeted analysis

## Abstract

A new analytical method based on the use of dispersive magnetic solid-phase extraction (DMSPE) is described for the preconcentration of capsaicin (CAP), dihydrocapsaicin (DCAP), and N-vanillylnonanamide (PCAP) from human serum samples. The influence of several experimental factors affecting the adsorption (nature and amount of magnetic material, adsorption time, and pH) and desorption (nature of solvent, its volume and desorption time) steps was studied. Among seven different nanomaterials studied, the best results were obtained using magnetic multiwalled carbon nanotubes, which were characterized by means of spectrometry- and microscopy-based techniques. Analyses were performed by ultra-high-performance liquid chromatography with quadrupole-time-of-flight mass spectrometry using electrospray ionization in positive mode (UHPLC-ESI-Q-TOF-MS). The developed method was validated by obtaining several parameters, including linearity (0.3–300 μg L^−1^ range), and limits of detection which were 0.1, 0.15, and 0.17 μg L^−1^ for CAP, DCAP, and PCAP, respectively. The repeatability of the method, expressed as relative standard deviation (RSD, *n* = 7), varied from 3.4 to 11%. The serum samples were also studied through a non-targeted approach in a search for capsaicinoid metabolites and related compounds. With this objective, the fragmentation pathway of this family of compounds was initially studied and a strategy was established for the identification of novel or less studied capsaicinoid-derived compounds.

**Graphical abstract:**

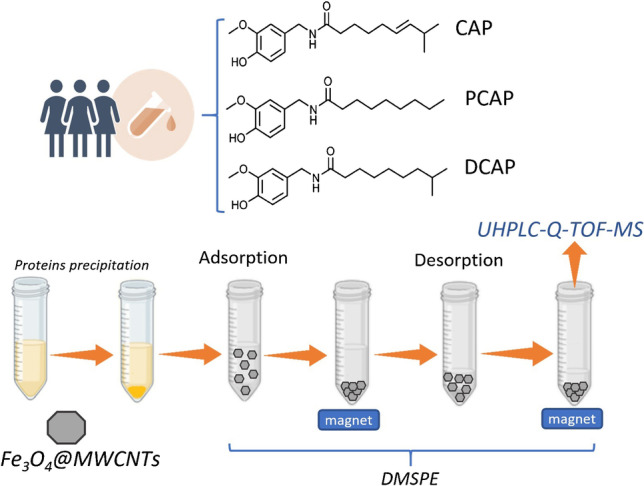

**Supplementary Information:**

The online version contains supplementary material available at 10.1007/s00216-023-04544-7.

## Introduction

Capsaicinoids are a group of alkaloids produced only within the *Capsicum* genus in the Solanaceae family. Their chemical structure includes a vanillyl group attached to an amide group and an alkyl chain, giving three different molecular sites with dipolar, hydrophilic, and lipophilic properties, endowing them with amphiphilic properties [1]. Capsaicin (CAP) and dihydrocapsaicin (DCAP) are the most abundant capsaicinoids in pepper fruits (80–90%), while N-vanillylnonanamide (PCAP) and other related compounds have substantially lower contents [1]. Capsaicinoids have shown anti-inflammatory, pain relief, body thermoregulation, anticancer, antioxidant, and antimicrobial activities [2]. CAP especially, when orally or topically administered, reduces inflammatory heat and the pain derived from rheumatoid arthritis or fibromyalgia [3]. CAP shows further benefits in the cardiovascular system not only for the prevention of hypertension but also for preventing myocardial infarction and coronary heart disease [3]. Another property of capsaicinoids, which has attracted attention in recent years, is their slimming effect, which could lead to high serum capsaicinoid levels that should be controlled because of the possible adverse effects on consumers. It has been reported that CAP and DCAP have the ability to inhibit the transcription of some genes responsible for the proteins stimulating thermogenesis or adipogenesis, so both play a crucial role in the regulation of obesity [3, 4]. However, it is known that the high toxic effect of CAP causes irritations in mucous membranes and tissues, leading to the establishment of a lethal dose of 50% (LD50) for humans of 0.5–5 g kg^−1^ [4].

On the other hand, even though there is no universal regulation, capsaicinoid-based products, considered as non-lethal weapons, can be used for personal defense and by law enforcement agencies, for example, in riot actions. Respiratory complications have been reported in asthmatics and even deaths after exposure to these products. For this reason and considering the easy access of the population to products for the treatment of very different health problems, such as overweight, the analysis of biological fluids has received increasing attention in judicial proceedings [1]. For such purposes, high sensitivity and accuracy are required for the analytical tools applied.

Several analytical techniques have been applied for the analysis of capsaicinoids, including gas chromatography [5] and liquid chromatography (LC), which has been by far the most widely preferred [6–18]. LC methods tend to use UV absorption spectrophotometry [6], fluorescence detection (FLD) [7–9], and mass spectrometry (MS) [10–18] as detection systems. Among the different available MS analyzers, triple quadrupole (QqQ) MS [10, 12–17] and high-resolution MS (HRMS) based on time of flight [18] have been satisfactorily applied for this purpose.

One of the main issues is the fact that the analytes are usually present at very low concentration levels, meaning that previous cleaning and preconcentration steps are needed. Traditionally, solid-phase extraction (SPE) [8, 12] and liquid-liquid extraction (LLE) [6, 10, 13–15] have been applied for isolating capsaicinoids from blood [10], urine [12, 18], serum [6, 15], plasma [13, 14], and tissues [10, 14]. During the last decades, microextraction methodologies have appeared in an attempt to overcome the disadvantages associated with LLE and SPE, including long application times, laborious procedures, and, for most cases, the high amounts of organic solvents used. Thus, miniaturized preconcentration techniques, such as liquid-phase microextraction (LPME) and those involving isolating the analyte into a solid phase, have achieved notable recognition and are currently widely applied in different areas because of the high extraction efficiency provided, and their rapidness and low organic solvent consumption, which make them suitable for being included within green analytical chemistry guidelines [19].

According to the literature, miniaturized sample treatments for the determination of capsaicinoid compounds in biological matrices are little used. Thus, dispersive liquid-liquid microextraction (DLLME) has been used for capsaicinoid preconcentration from human urine [18] and solid-phase microextraction from peppers and pepper sauces [5]. Analytical nanoscience using dispersive magnetic solid-phase extraction (DMSPE) has hardly been implemented for capsaicinoids, in particular for analysis of oil samples [9, 17]. This technique uses a magnetic material to isolate the compounds of interest by dispersing it through the sample solution, an approach offering many advantages over the use of the nanomaterial packaged into a cartridge [20]. As far as we know, the DMSPE methodology has not been previously applied for preconcentrating capsaicinoids from serum.

The novelty of this study was to develop an analytical method for the determination of CAP, DCAP, and PCAP in human serum based on DMSPE in combination with UHPLC-HRMS. This is also the first application of magnetic multiwalled carbon nanotubes (MWCNTs) for capsaicinoid isolation. The validated method was used in the analysis of ten serum samples of individuals treated with capsaicinoid-based topical creams. Additionally, a technological challenge is addressed consisting of a metabolomic study by means of a non-targeted approach and the establishment of a simple strategy for identification of novel or less studied capsaicinoid derivatives. Subsequently, this strategy was applied in the screening of capsaicinoid derivatives in serum samples. The proposed method opens the possibility of tracking human exposure to CAP, DCAP, and PCAP, which can enter the human body both intentionally and unintentionally, as well as through the study of derivative compounds.

## Materials and methods

### Reagents

Capsaicin (CAP, ≥ 95%), dihydrocapsaicin (DCAP, ≥ 85%), N-vanillylnonanamide (PCAP, ≥ 98%), and cyclohexanecarboxilic acid 3,4-dimethoxy-benzylamide (CADB, internal standard (IS)) were purchased from Sigma-Aldrich (St. Louis, MO, USA). Iron (III) chloride hexahydrate and ammonium iron (II) sulfate hexahydrate were obtained from PanReac AppliChem (Barcelona, Spain). Ammonia (solution 25% w/w) was provided from Scharlab S.L. (Barcelona, Spain). Formic acid was obtained from Merck KGaA (Darmstadt, Germany). Acetonitrile (AcN), methanol (MeOH), and ethanol (EtOH) were purchased from J.T. Baker (Deventer, The Netherlands).

Stock solutions of capsaicinoids and IS were prepared individually in MeOH at 500 mg L^−1^ and maintained at − 20 °C in glass vials. Intermediate and working solutions were prepared in water and maintained at 4 °C.

Multiwalled carbon nanotubes (MWCNTs), provided by Shenzhen Nanotech Port Co., Ltd. (Guangdong Sheng, China), have a specific surface area of 40–70 m^2^ g^−1^, average diameters in the 40–60-nm range, and average length higher than 5 μm. High-quality water was obtained using a Milli-Q system (Millipore, Bedford, MA, USA). For the synthesis of other magnetic materials different from the selected one, the following reagents (Sigma-Aldrich) were used: oleic acid (99%), silver nitrate (99.5%), (3-aminopropyl)triethoxysilane (98%, APTS), microcrystalline cellulose (powder, 20 μm), graphene oxide (GO, 2 mg mL^−1^ aqueous suspension), and pyrrole (98%).

### Instrumentation

A UHPLC system consisting of an Agilent 1290 Infinity II Series HPLC (Agilent Technologies, Santa Clara, CA, USA), equipped with a high-speed binary pump and an automated multisampler module, was used. The chromatograph was coupled to an Agilent 6550 Q-TOF Mass Spectrometer (Agilent Technologies, Santa Clara, CA, USA) by means of an Agilent Jet Stream Dual Electrospray (AJS-Dual ESI) interface. The software MassHunter Workstation Data Acquisition (Rev. B.08.00), from Agilent Technologies, was used to set the experimental parameters for UHPLC and Q-TOF.

Thermostated samples at 5 °C were injected (20 µL) onto an Omega Luna C18 (2.1 × 100 mm, 1.6 um) UHPLC column, at a flow rate of 0.4 mL min^−1^. The column was equilibrated at 25 °C. Isocratic elution was applied using mobile phase with a 50:50 A:B proportion for solvents A (0.1% v/v formic acid aqueous solution) and B (AcN containing 0.1% v/v formic acid). Under these experimental conditions, the compounds eluted in 7 min.

The ionization mode applied in the ESI source was positive in accordance with the literature consulted [13–18]. The nebulizer gas pressure was set to 40 psi, the drying gas flow was set to 16 L min^−1^ at a temperature of 150 °C, and the sheath gas flow to 12 L min^−1^ at 300 °C. Voltages for the capillary spray, fragmentor, nozzle, and octopole 1 RF Vpp were 4000, 360, 500, and 750 V, respectively. For MS scans, centroid data in the 50–500 *m/z* range were acquired in 2 GHz extended dynamic-range high-resolution mode with 2026 transients/spectrum, 250 ms/spectrum, and 4 spectra/s. Mass correction during the analysis was carried out using the reference mass at 121.0509. Extracted ion chromatograms (EICs) obtained from the protonated molecule of each compound, with a 0–5-ppm window, were used in the analyses both for identification and quantification purposes, in the latter case using chromatographic peak areas as the analytical signals. The adopted qualifier ions for each compound as well as the experimental and theoretical *m/z* values are shown in Table S1. The error for the experimental *m/z* values related to the theoretical ones was expressed as the difference between experimental and theoretical masses divided by the theoretical one and multiplied by 10^6^, in percentage terms. Error mass values of 2.5, 1.2, and 2.0 were obtained for CAP, DCAP, and PCAP, respectively.

For sample treatment purposes, an orbital stirrer (IKA-KS 130 Basic, Gottmadingen, Germany) and a refrigerated laboratory centrifuge MPW-150 R (MPW Med. Instruments, Warsaw, Poland) were used. Sample extracts were filtered before UHPLC analysis with Agilent 0.20-μm PTFE filter vials. Permanent magnets (Supermagnete, Gottmadingen, Germany) were blocks of Nd-Fe-B with 50 × 150 × 15 mm dimensions, 86 g weight, and 33 kg of strength.

### Synthesis and characterization of Fe_3_O_4_@MWCNTs

Magnetic nanomaterial was synthesized following the method previously published by Arroyo-Manzanares et al. [21]. Briefly, (NH_4_)_2_Fe(SO_4_)_2_·6H_2_O (0.85 g) and FeCl_3_·6H_2_O (0.42 g) were dissolved in 0.25 L of water; next, 0.5 g of previously purified MWCNTs was added. The suspension was ultrasonicated for 20 min at 50 °C. Fe_3_O_4_ was precipitated on the MWCNT walls by adding 25 mL of 8 mol L^−1^ ammonia solution dropwise while stirring. The mixture was maintained at 50 °C for 30 min for nanocomposite growth purposes. Once cooled at room temperature, the magnetic nanocomposite (Fe_3_O_4_@MWCNTs) was recovered by means of a permanent magnet and washed with purified water and EtOH. Finally, magnetic material was dried at 60 °C overnight, ground, and stored in glass recipients at room temperature.

For Fe_3_O_4_@MWCNT characterization, the hydrodynamic size of the nanomaterial was studied by dynamic light scattering (DLS) by means of a Malvern Zetasizer Nano ZS (Malvern Instruments Ltd, UK). The instrument was equipped with a He/Ne laser (4 mW) emitting at 633 nm, measuring cell, photomultiplier, and correlator. The scattering intensity at a 173° angle relative to the source (backscattering) was measured using an avalanche of photodiode detector set to room temperature. A general-purpose algorithm (integrated into the Malvern Zetasizer software) allowed the analysis of intensity autocorrelation functions with the aim of determining the distribution of the *z*-averaged translational diffusion coefficient of the particles (*D*_t_). The Stokes–Einstein equation relates the average *D*_t_ and the average hydrodynamic diameter (*d*_h_) of the particles:

$${d}_{\text{h}}=\frac{2{k}_{\text{B}}T}{{D}_{\text{t}}6\pi {\eta }_{\text{s}}}$$

with *k*_B_ as the Boltzmann constant, *T* as the absolute temperature, and *η*_s_ as the solvent viscosity. In this way, the radius of a compact sphere is defined by the *R*_h_ value, corresponding its translational diffusion coefficient to the average diffusion coefficient of the nanoparticles. The Nano ZS apparatus can also measure the zeta potential, providing information about the charge amount on the surface of the particles. Particles with zeta potentials out of the − 30 to + 30 mV range are stable, due the high electric repulsion, which prevents aggregation.

Transmission electron microscopy (TEM) was applied on the dispersed aqueous solution nanomaterial using a Philips Tecnai‐12 instrument operating at an accelerating voltage of 120 kV and equipped with a Megaview II camera to take the images. A suspension of the nanomaterial in water was prepared, and then, the water excess was removed using absorbent paper before taking the TEM images. The morphology and distribution of ferrite nanoparticles on MWCNTs were studied by TEM.

### Sample treatment

Ten serum samples were obtained from staff volunteers of the University of Murcia which had been treated with capsaicinoid-based creams and stored at − 20 °C until analysis. Prior to analysis, the samples were defrosted and 50 μL of CADB (0.25 mg L^−1^) and 1 mL of AcN were added to each 0.5-mL serum sample before being centrifuged at 4000 rpm and 10 °C for 5 min to precipitate the protein. The supernatant was collected and taken up to 10 mL with water.

In a 15-mL conical plastic tube, 50 mg of Fe_3_O_4_@MWCNTs was weighed. Next, 10 mL of sample solution (containing 0.5 mL of serum) was added to the solid magnetic material and the mixture was stirred by orbital shaking for 15 min at 640 rpm. Then, a magnetic nanocomposite enriched with the analytes was attracted with an external magnet in order to eliminate the supernatant solution. Capsaicinoids were desorbed in 1.5 mL of AcN, while the mixture was being stirred by orbital shaking at 640 rpm for 5 min. In a final step, the organic extract was evaporated using a nitrogen steam, reconstituted by adding 50 µL of AcN and filtered using PTFE filter vials. A 20-μL volume of the resultant preconcentrated phase was injected into the chromatographic system for analysis.

For recovery studies, two serums were fortified at two concentration levels (5 and 50 μg L^−1^) by adding 0.01 and 0.1 mL of a 0.25-μg mL^−1^ standard solution, manually stirred for a few seconds and left to stand for 1 h before applying the above-described procedure. Each analysis was performed in triplicate.

This study has a favorable report (ID: 2908/2020) from the Research Ethical Committee of the University of Murcia.

## Results and discussion

### Optimization of DMSPE

In order to simplify the sample matrix before its preconcentration by DMSPE, two different procedures were assayed, both including a protein precipitation step with AcN. First, 1 mL of AcN was added to 500 µL of serum (spiked with the analytes at 0.1 mg L^−1^) and the mixture was centrifuged for 4 min at 3000 rpm. Next, the supernatant was recovered, made up to 10 mL with water, and preconcentrated by DMSPE. In the second procedure, in order to prevent possible lipid interferences, 1 mL of hexane was added to the supernatant and the homogenized mixture was centrifuged for another 4 min at 3000 rpm. The lower phase was recovered, made up to 10 mL with water, and submitted to DMSPE. These experiments were carried out in duplicate. Because no significant differences in terms of capsaicinoid sensitivity and chromatographic profiles were observed, the first approach was selected. The volume of serum was fixed at the maximum sample availability (0.5 mL).

To obtain the highest extraction efficiency for capsaicinoids, the influence of the different variables affecting both the adsorption and desorption steps in the DMSPE procedure was evaluated using the supernatant obtained in the treatment of 500 μL of serum (spiked with the analytes at 0.1 mg L^−1^) with 1 mL AcN and diluted to 10 mL with water. Since the nature of the sorbent magnetic material is determinant in preconcentration efficiency, several magnetic materials were synthesized following previously published procedures: oleic acid [22], silver [23], (3-aminopropyl)triethoxysilane (APTS) [24], cellulose [25], GO [26], polypyrrole (PPy) [26], MWCNTs@PPy [26], and MWCNTs [21], all of them magnetized with Fe_3_O_4_. Possible interactions between capsaicinoids and oleic acid sorbent due to long hydrocarbon chains contained in both structures led us to try Fe_3_O_4_@oleic acid. In the same way, the probable coordination interaction between amino groups of the analytes with Ag nanoparticles [27] present in Fe_3_O_4_@Ag sorbent was checked, as well as the good affinity previously provided for APTS sorbent toward chemical structures containing carbonyl, amino, and benzene groups [24, 28]. The potential biodegradability of cellulose was the reason for studying this sorbent. Previous DMSPE studies for capsaicinoids using GO [17] led us to compare its efficiency with that of MWCNTs, whose affinity for very different types of organic compounds has been proven, as well as the polymeric sorbent PPy and PPy/MWCNT combination

**Fig. 1:**
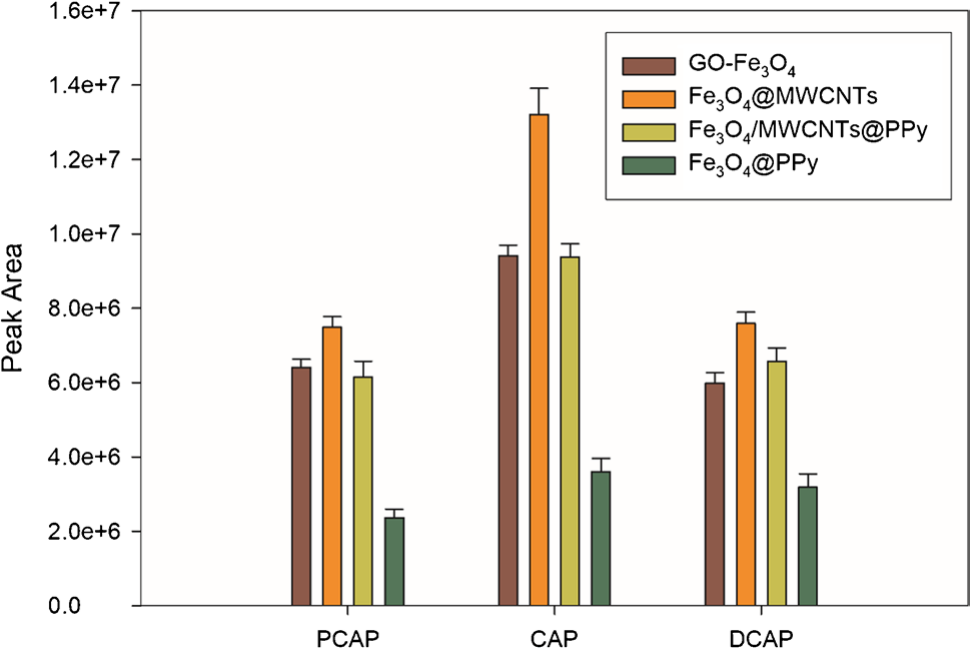
Effect of the nature of the DMSPE functionalized magnetic sorbent on the preconcentration of capsaicinoids. Vertical bars indicate standard deviation for *n* = 2

Figure 1 shows the extraction capacity of the different nanomaterials studied, using 20 mg in each case. The rest of the experimental conditions were as follows: 30 min adsorption time while orbitally shaking the mixture at 640 rpm, and desorption of the analytes by orbital shaking of the enriched magnetic adsorbent at 640 rpm with 1.5 mL of AcN for 15 min. Finally, 20 µL of the organic extract was injected into the UHPLC-Q-TOF-MS system. No preconcentration was observed in the case of Fe_3_O_4_@cellulose, Fe_3_O_4_@Ag, Fe_3_O_4_@APTS, and Fe_3_O_4_@oleic acid. As shown in Fig. 1, Fe_3_O_4_@MWCNTs provided the best results, which may be attributed to hydrophobic interactions between the capsaicinoids and the MWCNTs, specifically *π*-*π* interactions between the benzene rings of the analytes and the bulk systems on the MWCNT surface, as well as those of the hydrocarbon chain of the target compounds. Thus, Fe_3_O_4_@MWCNT was the magnetic material selected.

**Fig. 2:**
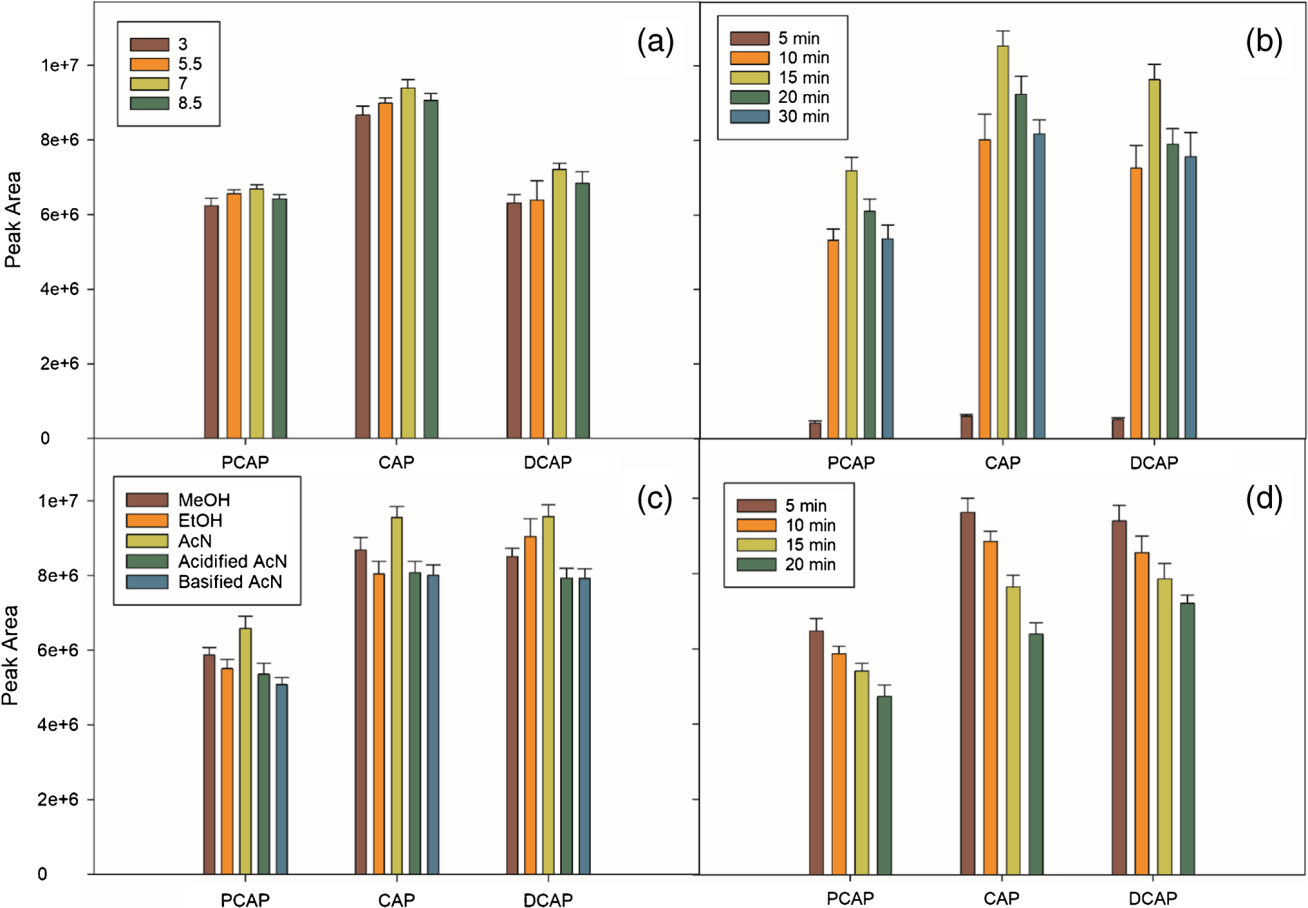
Effect of **a** pH of the aqueous phase, **b** adsorption time, **c** desorption solvent nature, and **d** desorption time on the DMSPE efficiency. Vertical bars indicate standard deviation for *n* = 2

When the influence of the pH of the aqueous phase was evaluated in the 3-to-8.5 range using formic acid and ammonia, the results obtained were very similar in all cases but slightly higher in the analytical signals for pH 7 (Fig. 2a), which was the value selected. The adsorption time was studied in the 5–30-min range. As shown in Fig. 2b, the adsorption capacity notably increased up to 15 min, and then suffered a slight decrease at longer times. Consequently, the adsorption time was set to 15 min.

The magnetic material enriched with the analytes was contacted with 1.5 mL of the different desorption solvents (MeOH, EtOH, and AcN) over a period of 5 min, while submitting the mixture to orbital shaking at 640 rpm. AcN provided the highest sensitivity. The effect of pH was studied at 4 and 9, using formic acid and ammonia, respectively. As shown in Fig. 2c, AcN with no pH adjustment gave the best results for the three capsaicinoids studied and was therefore selected. Desorption times of 5, 10, 15, and 20 min were studied (Fig. 2d). The best results were obtained for the shortest time assayed, the analytical signals decreasing up to 20 min.

**Fig. 3:**
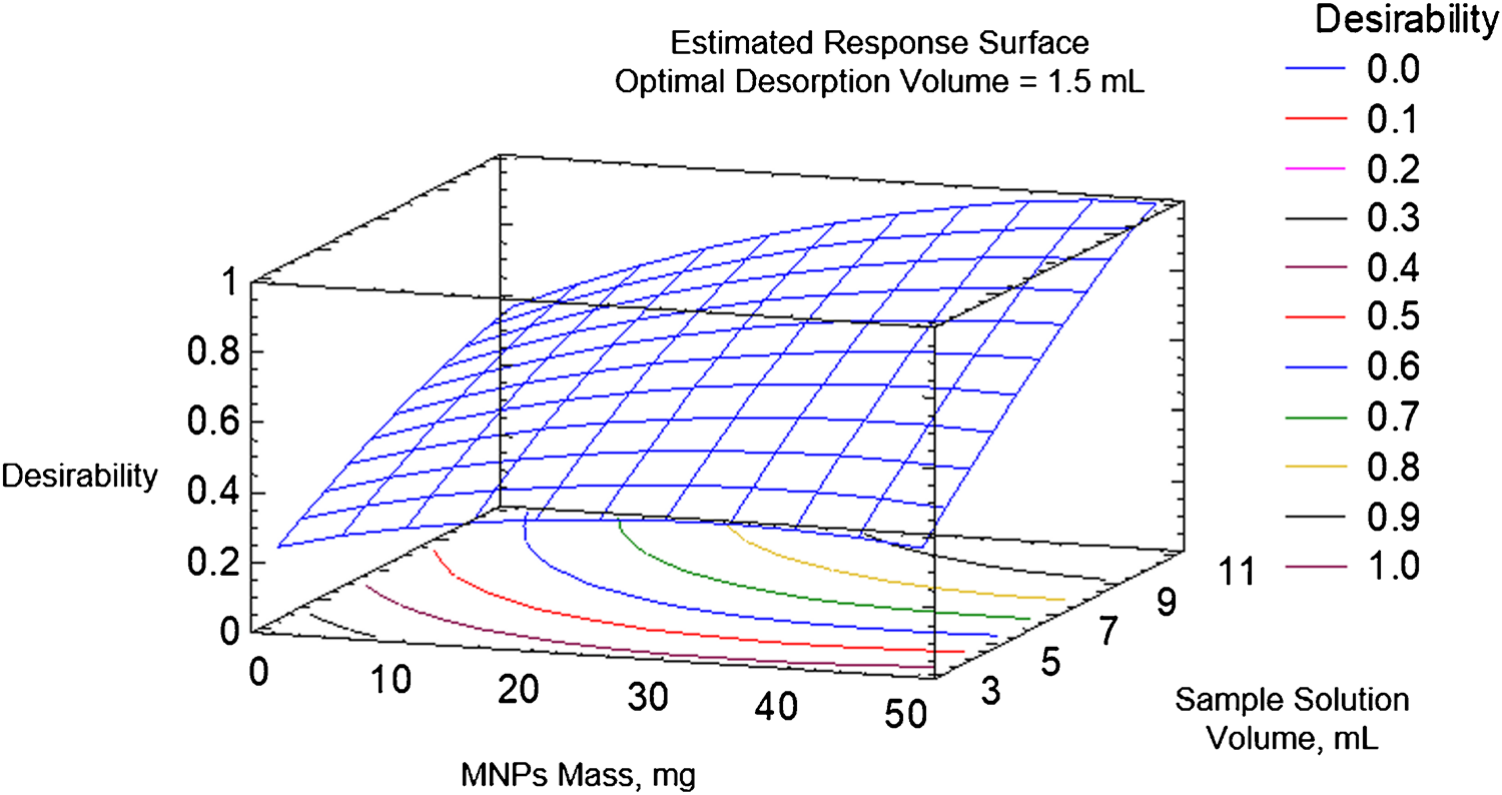
Response surface obtained for the optimization of sample solution volume, magnetic nanocomposite mass, and desorption solvent volume in DMSPE

Sample solution volume, Fe_3_O_4_@MWCNT mass, and desorption solvent volume were optimized by using a multivariate study based on a central composite design (CCD, 2^3+star), applying sample volumes in the 3–10-mL range, nanocomposite masses of between 10 and 50 mg and desorption solvent volumes of 1.5 to 5 mL. The results obtained showed that the optimal conditions corresponded to 50 mg of Fe_3_O_4_@MWCNTs maintained in contact with 10 mL of sample solution and desorbed in 1.5 mL of AcN. The response surface obtained is shown in Fig. 3. Briefly, the results indicated that an increase in Fe_3_O_4_@MWCNT mass and sample volume has a positive effect on the analysis, while increasing the volume of AcN in the desorption step produces the opposite effect.

Finally, with the aim of increasing the method’s sensitivity, evaporation of the AcN phase obtained in the DMSPE desorption step and reconstitution in a lower AcN volume was tested. A reconstitution volume of AcN of 50 µL was selected and allowed a total and precise recovery of the dried residue. When lower volumes were tested, recovery of the extract was poorer, and as higher volumes would lead to a dilution effect, they were not assayed.

### Characterization of magnetic material

The Fe_3_O_4_@MWCNTs nanomaterial selected for the proposed DMSPE procedure has previously been characterized by Arroyo-Manzanares et al. [21] by means of the techniques energy-dispersive X-ray spectrometry, X-ray diffraction, scanning electron microscopy (SEM), and Fourier transform infrared spectrometry. Other studies have since been carried out to obtain additional information about the magnetic nanomaterial and to improve its characterization. For this, DLS and TEM techniques were applied.

**Fig. 4:**
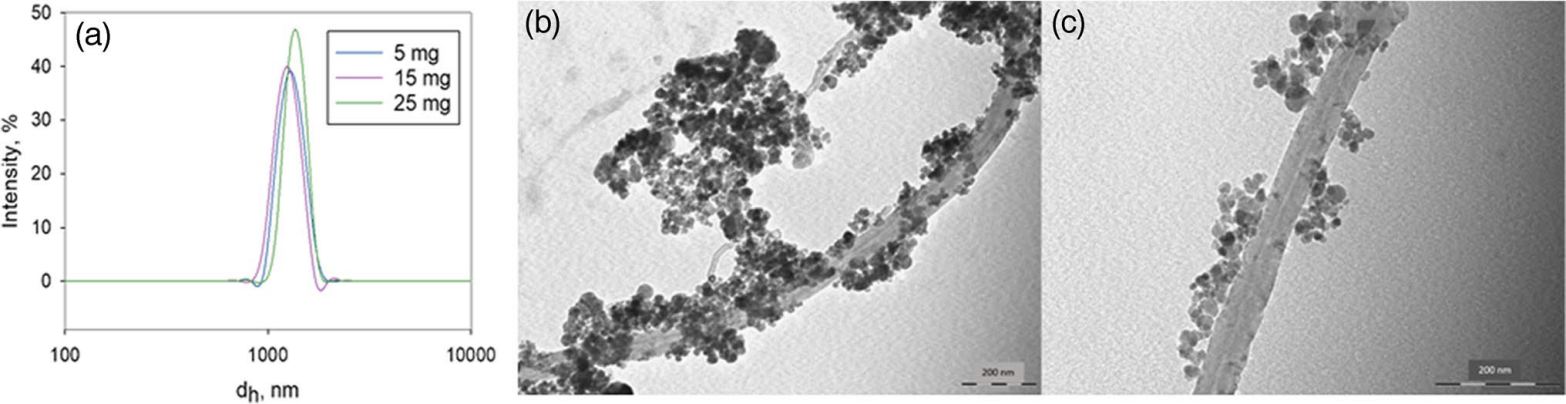
**a** Size distribution of Fe_3_O_4_@MWCNT suspension at different concentrations. **b** and **c** TEM images of Fe_3_O_4_@MWCNTs with a scale of 200 nm

DLS measurements were carried out by adding Fe_3_O_4_@MWCNT masses of 5, 15, and 25 mg to 2 mL of water and applying a single cycle of 16 runs. No equilibrating time was needed. Under these conditions, the hydrodynamic diameter (*d*_h_) values obtained were 1316, 1245, and 1372 nm for each amount of nanomaterial measured, which means an average diameter of 1311 nm (Fig. 4a). *Z* potential values were obtained under the same experimental conditions as for hydrodynamic size and were − 4.52, 2.74, and 0.252 mV. These values show that the nanocomposite surface charge is close to zero, pointing to the absence of electrostatic repulsion forces whose function is to prevent aggregation. Thus, Fe_3_O_4_@MWCNT tends to aggregate, and, for this reason, continuous stirring is recommended during the DMSPE adsorption step.

The TEM images provided in Fig. 4b, c correspond to the 200-nm scale showing two different regions of the dispersed material, allow to appreciate that Fe_3_O_4_ microspheres (35–95-nm diameters) were randomly immobilized onto the outside walls of the MWCNTs, as has previously been observed by SEM [21]. Moreover, the TEM images showed that the MWCNTs do not tend to aggregate, while this effect is observed in the case of Fe_3_O_4_ microspheres.

### Method validation and analysis of human serum samples

The developed method was validated in terms of linearity, limits of detection (LOD) and quantification (LOQ), accuracy, and precision. Calibration curves for the capsaicinoids were obtained by the IS method using CADB (25 μg L^−1^), which had been shown not to be contained in the analyzed samples and to have similar chromatographic and chemical behaviors as the analytes. Under the selected conditions, the retention time for CADB was 1.72 min. The linear regression analysis was carried out by plotting the ratio of the peak area for each analyte to that of the IS peak area *versus* analyte concentration for eight levels, performing the assays for each level twice. The analytes showed a linearity range of 0.3–300 μg L^−1^, depending on the compound (Table 1). Correlation coefficient values were in all cases higher than 0.996.

**Table 1:** Analytical characteristics of the proposed method

Compound	*t*_R_ (min)	Linearity (μg L^−1^)	*R*^2^	LOD^a^ (μg L^−1^)	RSD^b^ (%)	
					5 μg L^−1^	50 μg L^−1^
PCAP	3.502	0.6-300	0.998	0.17	11	3.7
CAP	3.536	0.3-300	0.999	0.10	11	3.4
DCAP	5.009	0.5-300	0.996	0.15	9.5	5.8

^a^Calculated for a signal-to-noise ratio of 3

^b^*n* = 7 (intraday)

Since the presence of certain substances coextracted with the analytes from the samples may affect the response of the detection system, the possible matrix effect was studied by comparison of the slopes obtained through aqueous calibration with those obtained by applying the standard additions method for two different serum samples (Table S2). For this purpose, an ANOVA statistical test was used. The absence of matrix effect was confirmed because the “*p*” values were higher than 0.05 in all cases at the 95% confidence level. Therefore, aqueous calibration was applied for sample quantification purposes.

The sensitivity of the method was evaluated by calculating LODs and LOQs for signal-to-noise ratios of 3 and 10, respectively. The values obtained for the LODs are shown in Table 1 and were in the 0.1–0.17 range, being between 0.33 and 0.57 μg L^−1^ the corresponding LOQs. Method repeatability was studied as the relative standard deviation (RSD) for a series of seven consecutive analyses of a serum sample fortified at 5 and 50 μg L^−1^. RSD values were between 9.5–11% and 3.4–5.8% for the low and the high spiking level, respectively (Table 1).

Because no reference materials were available, recovery studies were carried out to test the method’s accuracy. Thus, two different samples were fortified at 5 and 50 μg L^−1^ and their analysis provided recovery values in the 97.0–99.4 and 94.6–96.9% for the lowest and the higher concentration, respectively.

A comparison of the developed method with others previously published dealing with the determination of capsaicinoid compounds in serum and plasma samples is presented in Table 2. As can be observed, only one previous study [6] deals with the analysis of human serum, the rest focusing on animal serum. Moreover, this is the first method involving HRMS, permitting therefore a metabolomic search for exact mass measurements of possible capsaicinoid metabolized and related compounds. Thus, the proposed method represents a methodological improvement with respect to the use of low-resolution MS. As regards method sensitivity, lower LODs were obtained than those obtained by conventional sample treatments such as SPE with HPLC-FLD analysis [8] and LLE with HPLC-UV analysis [6], in the latter case using a sample volume four times greater than the proposed method. Low LOD values were provided by tandem MS with QqQ analyzer [13–15], but HRMS permitted searching for non-targeted compounds. Moreover, LLE-based sample treatments use toxic organochlorine solvents [14, 15] or the very volatile solvent methyl tert-butyl ether [13] that should be handled under conditions in which the method precision is not compromised. When comparing DMSPE with the SPE technique, the dispersion of the extractant phase into the sample solution allows higher analyte recovery to be obtained. In addition, the reusability of the nanoparticles is a major advantage over the packaged extractant phases used in SPE. With respect to method precision, the DMSPE sample treatment here proposed provides repeatability values similar to those obtained by other methodologies based on liquid-phase extraction. As regards the two previous DMSPE published procedures for capsaicinoid determination in oils, of note is the similar sensitivity here achieved even through the use of detection systems of higher sensitivity, based on tandem MS with QqQ [17], or specific sorbents such as molecularly imprinted polymers [9].

**Table 2:** Comparison of the proposed method with others previously published for capsaicinoid determination in serum and plasma samples

Analytes	Sample nature and amount	Sample treatment and total organic solvent consumption	Instrumental measurement	LOD (µg L^−1^)	RSD^b^ (%)	Ref.
CAP, DCAP	Human serum and plasma (2 mL)	LLE with 2 mL MIBK. 0.2 mL AcN for dried extract reconstitution	HPLC-UV (205 nm)	2.6, 3.8	9.1, 7.4	[6]
CAP, DCAP	Dog plasma (0.5 mL)	Protein precipitation (0.5 mL AcN) and SPE (3 mL MeOH). 50 µL AcN for dried extract reconstitution	HPLC-FLD (230; 323 nm)	2	3.0, 0.4	[8]
CAP, DCAP	Equine plasma (1 mL)	LLE with 5 mL methyl tert-butyl ether. 60 µL MeOH for dried extract reconstitution	UHPLC-QqQ-MS/MS with ESI+	0.0005	1.9–18.1	[13]
CAP, DCAP	Rabbit plasma (0.1 mL)	LLE with 1 mL N-hexane:CH_2_Cl_2_:isopropanol (100:50:5). 90 µL AcN for dried extract reconstitution	HPLC-QqQ-MS/MS with ESI+	0.125^a^	5.6–7.5	[14]
CAP, DCAP	Horse serum (0.2 mL)	LLE with 1(x5) mL CH_2_Cl_2_. 25 µL MeOH for dried extract reconstitution	UHPLC-QqQ-MS/MS with ESI+	0.0005, 0.001^a^	Not provided	[15]
CAP, DCAP, and PCAP	Human serum (0.5 mL)	Protein precipitation with 1 mL of AcN and DMSPE (1.5 mL AcN). 50 µL AcN for dried extract reconstitution	UHPLC-Q-TOF-MS with ESI+	0.10, 0.15, 0.17	3.4–11	This work

^a^Lower limit of quantitation

^b^Repeatability studies

Ten human serum samples from patients previously treated with capsaicinoid-based creams were analyzed under the finally selected conditions for the DMSPE method with UHPLC-Q-TOF-MS. Neither CAP, DCAP, nor PCAP was found in any of the samples, at least above their respective LODs. Figure S1 shows the EICs obtained by applying the developed method for a serum sample fortified at 5 μg L^−1^.

The analysis of the serum of animals topically exposed to these compounds has been previously considered. Thus, Wang et al. [14] concluded a very low absorption in the systemic circulation of rabbits because high levels were detected in the skin and negligible values in serum. In the study of Kuzma et al. [8], it is noteworthy that even considering the ideal situation of 100% bioavailability and 100% recovery, the analytes were not detected in dog serum. In fact, these results were in accordance with earlier studies demonstrating that intestinal accumulation and hepatic metabolism limit the systemic pharmacological effects of capsaicinoids. You et al. [13] and Zak et al. [15] analyzed horse plasma using HPLC-QQQ-MS, detecting CAP contents at very low concentrations. Consequently, our procedure provides new data confirming that detectable levels of capsaicinoids also do not appear in human serum after topical exposure, although they could appear after other types of exposure.

### Non-targeted study for identification of capsaicinoid-derived compounds

Prior to the non-targeted study and in order to establish a route for the identification of capsaicinoid-derived compounds, the fragmentation of CAP, DCAP, and PCAP was studied.

**Fig. 5:**
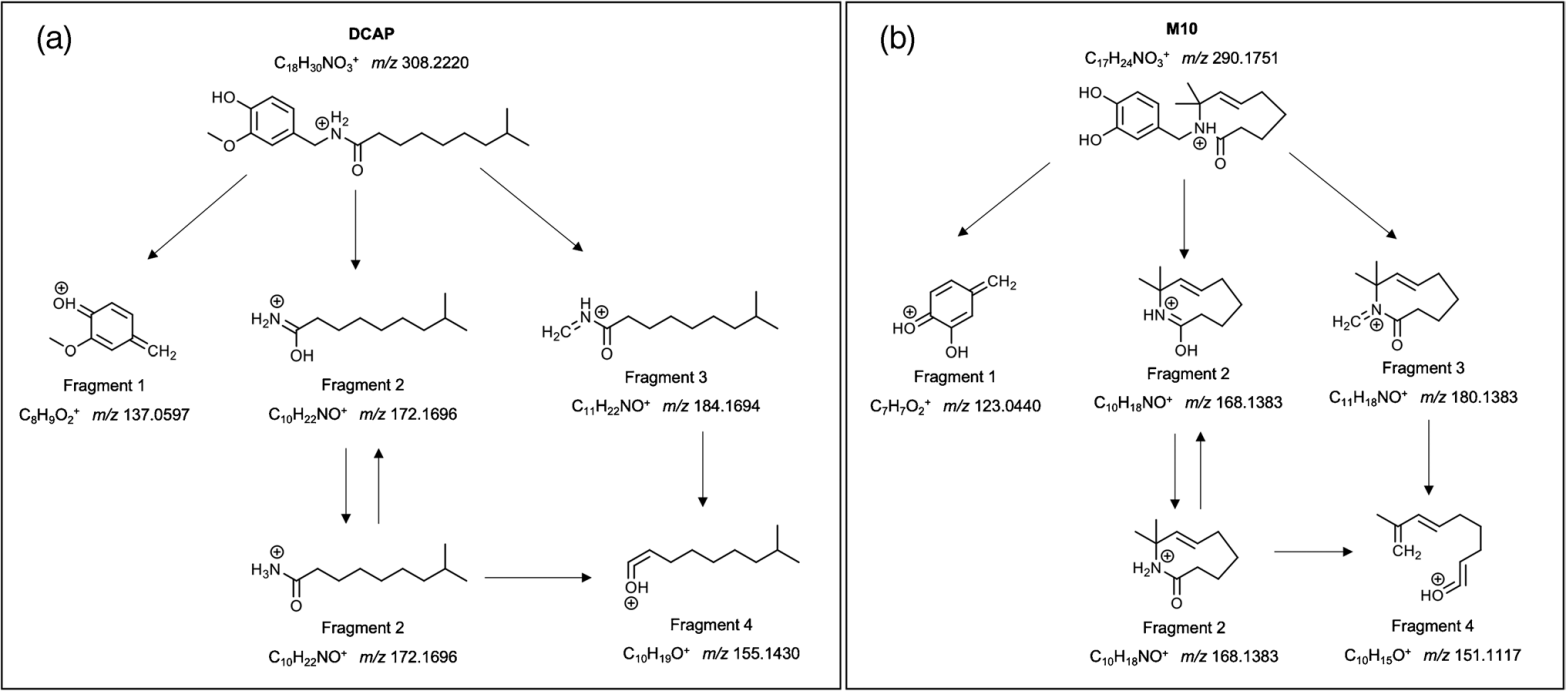
Fragmentation pathway of **a** DCAP and **b** M10

Figure S2 shows the full HRMS/MS spectrum of the precursor DCAP (*m/z* 308). It is characterized mainly by four fragment ions. The parent ion with *m/z* 308.2224 (error 1.2 ppm) underwent a cleavage of the C7-N8 position yielding the ion *m/z* 137.0595 ion assigned as C_8_H_9_O_2_^+^ (error − 1.5 ppm), which involves a rearrangement of the double bonds of the aromatic ring structure, and the ion with *m/z* 172.1691 corresponding to the acyl chain that results from removing the aromatic ring (C_10_H_22_NO^+^, error − 2.8 ppm). An ion at *m/z* 155.1426 (C_10_H_17_O^+^, − 2.8 ppm) was also observed corresponding to the loss of NH_3_ from *m/z* 184.1699. Finally, the ion with *m/z* 184.1699 was also observed and assigned to C_11_H_22_NO^+^ (error 1.7 ppm). This ion corresponds to the loss of the aromatic ring from the precursor DCAP and underwent subsequent loss of –CH_2_NH yielding again ion at *m/z* 155.1426. The fragmentation pathway of DCAP is shown in Fig. 5a. CAP and DCAP showed the same fragmentation pathway, which is in accordance with what is described in the bibliography [29, 30].

To date, 31 compounds of the CAP family or derivatives of their metabolization have been described. Supplemental Table S3 lists the structures of all these derivatives together with their molecular formula and exact mass.

The M1-M19 compounds are derived from the CAP metabolic pathway. Most of them have the same structure based on an aromatic ring and the acyl chain, so it is expected that they follow the same fragmentation pathway as CAP, DCAP, and PCAP. However, compounds M1, M10, M16, and M17 replace the chain with a cyclic structure bonding to the –NH group. The fragmentation of these compounds has also been evaluated using HRMS/MS spectra and information available in the literature [29, 30]. As can be seen, fragmentation follows a similar pathway to that of DCAP. Figure 5 shows the proposed fragmentation pathway for the M10 compound. Like DCAP, the parent ion with *m/z* 290.1751 underwent a cleavage of the C7-N8 position yielding the ion *m/z* 123.0440 (C_7_H_7_O_2_^+^), corresponding to the aromatic ring, and *m/z* 168.13829 (C_10_H_18_NO^+^), corresponding to the acyl cyclic, which subsequently produced ion at *m/z* 151.1117 (C_10_H_15_O^+^) by loss of NH_3_. Finally, the ion corresponding to the loss of the aromatic ring from the precursor M10 was also detected (*m/z* 180.13829, C_11_H_18_NO^+^).

**Fig. 6:**
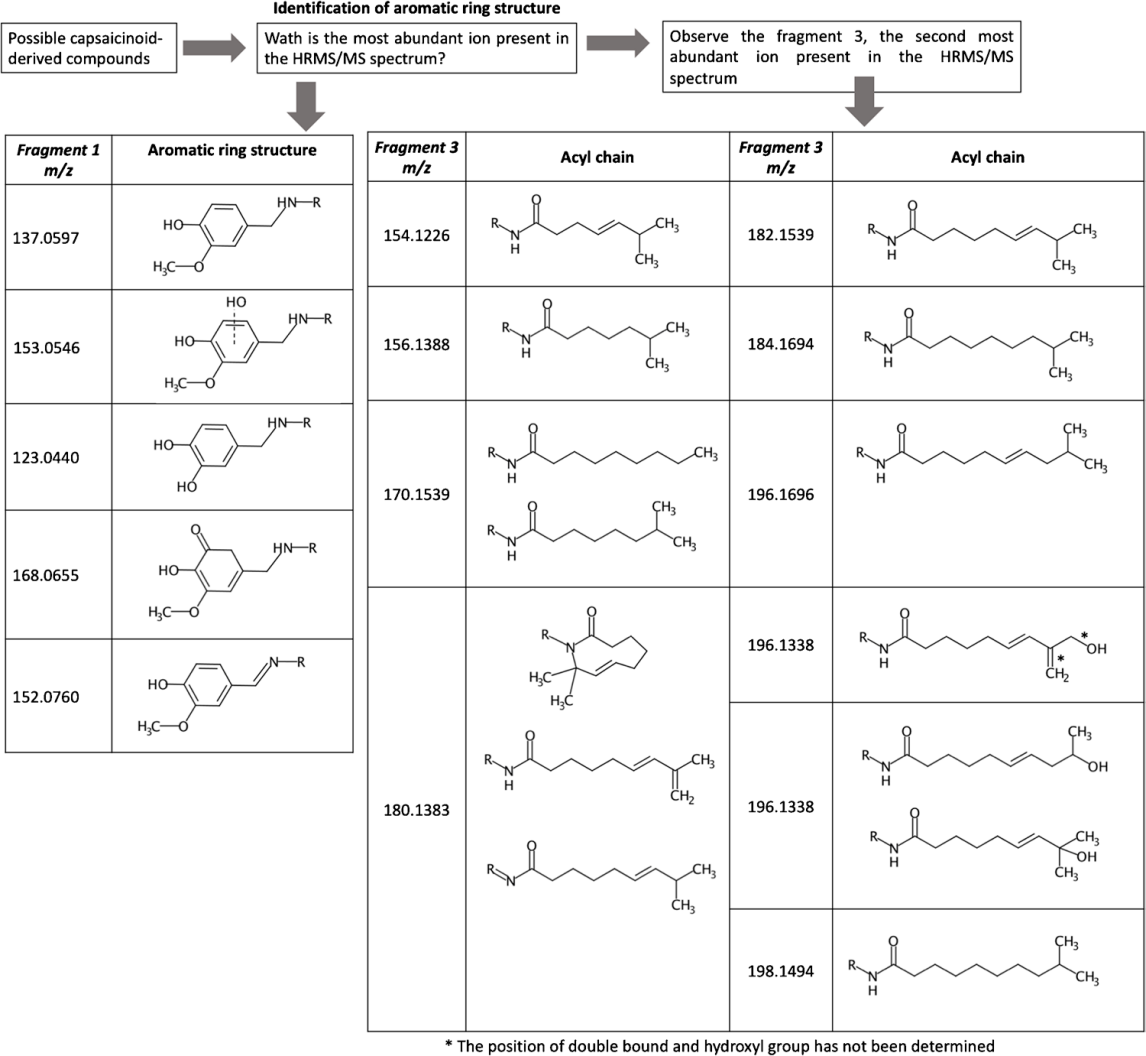
Proposed strategy for identification of novel capsaicinoid-derived compounds

Therefore, it can be concluded that four major ions dominate in the fragmentation spectra of capsaicinoid-derived compounds (Table 3). Table S3 shows the exact masses and molecular formulas of the four main fragmentation ions for all the derivatives described in the bibliography. Based on this information, a strategy for the identification of novel or less studied capsaicinoid-derived compounds was proposed (Fig. 6).

**Table 3:** Predominant ions in the HRMS/MS spectra of capsaicinoid-derived compounds

Compound	[M+H]^+^		Fragment 1		Fragment 2		Fragment 3		Fragment 4	
	Molecular formula	*m/z*	Molecular formula	*m/z*	Molecular formula	*m/z*	Molecular formula	*m/z*	Molecular formula	*m/z*
CAP	C_18_H_28_NO_3_^+^	306.2064	C_8_H_9_O_2_^+^	137.0597	C_10_H_20_NO^+^	170.1539	C_11_H_20_NO^+^	182.1539	C_10_H_17_O^+^	153.1274
DCAP	C_18_H_30_NO_3_^+^	308.2220	C_8_H_9_O_2_^+^	137.0597	C_10_H_22_NO^+^	172.1696	C_11_H_22_NO^+^	184.1694	C_10_H_19_O^+^	155.1430
PCAP	C_17_H_28_NO_3_^+^	294.2064	C_8_H_9_O_2_^+^	137.0597	C_9_H_20_NO^+^	158.1539	C_10_H_20_NO^+^	170.1539	C_9_H_17_O^+^	141.1274
Nordihydrocapsaicin (NDCAP)	C_17_H_28_NO_3_^+^	294.2065	C_8_H_9_O_2_^+^	137.0597	C_9_H_20_NO^+^	158.1539	C_10_H_20_NO^+^	170.1539	C_9_H_17_O^+^	141.1274
Homocapsaicin (HCAP)	C_19_H_31_NO_3_^+^	320.2220	C_8_H_9_O_2_^+^	137.0597	C_11_H_22_NO^+^	184.1694	C_12_H_22_NO^+^	196.1696	C_11_H_19_O^+^	167.1429
Homodihydrocapsaicin (HDCAP)	C_19_H_32_NO_3_^+^	322.2383	C_8_H_9_O_2_^+^	137.0597	C_11_H_24_NO^+^	186.1857	C_12_H_24_NO^+^	198.1859	C_11_H_20_O^+^	169.1592
Nornordihydrocapsaicin (NDCAP)	C_16_H_26_NO_3_^+^	280.1907	C_8_H_9_O_2_^+^	137.0597	C_8_H_18_NO^+^	144.1388	C_9_H_18_NO^+^	156.1388	C_10_H_15_O^+^	151.1117
Nornorcapsaicin (NCAP)	C_16_H_24_NO_3_^+^	278.1751	C_8_H_9_O_2_^+^	137.0597	C_8_H_16_NO^+^	142.1226	C_9_H_16_NO^+^	154.1226	C_10_H_13_O^+^	149.0961
M1	C_18_H_26_NO_3_^+^	304.1907	C_8_H_9_O_2_^+^	137.0597	C_10_H_18_NO^+^	168.1383	C_11_H_18_NO^+^	180.1383	C_10_H_15_O^+^	151.1117
M2	C_18_H_28_NO_4_^+^	322.2013	C_8_H_9_O_2_^+^	137.0597	C_10_H_20_NO_2_^+^	186.1489	C_11_H_20_NO_2_^+^	198.1494	C_10_H_17_O_2_^+^	169.1223
M3	C_18_H_28_NO_4_^+^	322.2013	C_8_H_9_O_2_^+^	137.0597	C_10_H_20_NO_2_^+^	186.1489	C_11_H_20_NO_2_^+^	198.1494	C_10_H_17_O_2_^+^	169.1223
M4	C_18_H_26_NO_3_^+^	304.1913	C_8_H_9_O_2_^+^	137.0597	C_10_H_18_NO^+^	168.1383	C_11_H_18_NO^+^	180.1383	C_10_H_15_O^+^	151.1117
Isomers M5 and M7	C_18_H_28_NO_4_^+^	322.2019	C_8_H_9_O_3_^+^	153.0546	C_10_H_20_NO^+^	170.1539	C_11_H_20_NO^+^	182.1539	C_10_H_17_O^+^	153.1274
M6	C_17_H_26_NO_3_^+^	292.1913	C_7_H_7_O_2_^+^	123.0440	C_10_H_20_NO^+^	170.1539	C_11_H_20_NO^+^	182.1539	C_10_H_17_O^+^	153.1274
M8	C_18_H_28_NO_4_^+^	322.2019	C_8_H_10_NO_3_^+^	168.0655	C_10_H_20_NO^+^	170.1539	C_11_H_20_NO^+^	182.1539	C_10_H_17_O^+^	153.1274
M9	C_18_H_26_NO_3_^+^	304.1907	C_8_H_10_NO_2_^+^	152.0760	C_10_H_18_NO^+^	168.1383	C_11_H_18_NO^+^	180.1383	C_10_H_15_O^+^	151.1117
M10	C_17_H_24_NO_3_^+^	290.1751	C_7_H_7_O_2_^+^	123.0440	C_10_H_18_NO^+^	168.1383	C_11_H_18_NO^+^	180.1383	C_10_H_15_O^+^	151.1117
M11	C_17_H_24_NO_3_^+^	290.1751	C_7_H_7_O_2_^+^	123.0440	C_10_H_18_NO^+^	168.1383	C_11_H_18_NO^+^	180.1383	C_10_H_15_O^+^	151.1117
M12	C_17_H_26_NO_4_^+^	308.1856	C_7_H_7_O_2_^+^	123.0440	C_10_H_20_NO_2_^+^	186.1489	C_11_H_20_NO_2_^+^	198.1494	C_10_H_17_O_2_^+^	169.1223
Isomers M13 and M14	C_18_H_28_NO_5_^+^	338.1962	C_8_H_9_O_3_^+^	153.0546	C_10_H_20_NO_2_^+^	186.1489	C_11_H_20_NO_2_^+^	198.1494	C_10_H_17_O_2_^+^	169.1223
M15	C_18_H_26_NO_4_^+^	320.1856	C_8_H_9_O_2_^+^	137.0597	C_10_H_18_NO_2_^+^	184.1338	C_11_H_18_NO_2_^+^	196.1338	C_10_H_15_O_2_^+^	167.1082
Isomers M16 and M1, isomers M18 and M19	C_18_H_26_NO_4_^+^	320.1856	C_8_H_9_O_3_^+^	153.0546	C_10_H_18_NO^+^	168.1383	C_11_H_18_NO^+^	180.1383	C_10_H_15_O^+^	151.1117

Fragment 1 is the major ion in the HRMS/MS spectrum and allows the identification of the aromatic ring present in the structure. The ion *m/z* 137.0597 corresponds to the structure of CAP, and therefore is present in all those compounds that share part of this structure with CAP. The ion *m/z* 153.0546 implies a hydroxylation on the aromatic ring and *m/z* 123.0440, O-demethylation. Compounds M8 and M9 presented ions *m/z* 168.0655 and 152.0760, respectively, indicating that this aromatic ring underwent fragmentation and retained the –NH group in its structure. This is due in the case of M8 to the fact that it shows an additional C=O bond in its aromatic ring, which avoids the rearrangement of the double bonds. In the case of M9, this compound has a double bond at the –NH group, which also makes it difficult to break the C7–N8 bond. Table S4 shows the structure of fragment 1 of all the described compounds of the CAP family. The observation of the second most abundant ion of HRMS/MS allows the identification of the rest of the structure of the capsaicinoid-derived compounds, as can be seen in Fig. 6.

The strategy above-described was applied to detect possible derivatives or compounds of capsaicinoid metabolization in the analyzed samples. With this purpose, data acquired using an all-ion method were processed following a non-targeted approach. For data processing, the free MS-DIAL software was used, and a metabolomics-based methodology was applied. Tolerance masses of 0.01 and 0.025 Da were used for MS1 and MS2, respectively, detecting a peak with a minimum height of 1000. The deconvolution was carried out using a sigma window value of 0.5. For peak identification, a database was created with the information of the compounds described in Table S3, allowing an accurate mass tolerance of 0.01 Da and an identification score cutoff of 85%. In order to increase the monitoring range, the most important conjugations resulting from biotransformation in the body were considered and glutathione (GSH), glucuronide (G), or sulfate conjugates and CAP-dimer were also included in the database. After identification, sample alignment was performed using a retention time and MS1 tolerances of 0.05 min and 0.015 Da, respectively.

Applying this strategy, the compounds CAP, DCAP, and PCAP in the fortified samples were correctly identified; however, none of their possible metabolites or derivatives were detected in the samples analyzed. To ensure that the samples did not contain other derivatives not included in the database, the fragment 1 described in Fig. 6 was used to screen for this family of compounds by observing their EICs of the low- and high-voltage fragmentation spectra in the MassHunter software. The absence of capsaicinoid derivatives in the analyzed samples was confirmed.

## Conclusions

A magnetic nanomaterial based on ferrite and MWCNTs applying DMSPE offers excellent performance to isolate CAP, DCAP, and PCAP from human serum samples with a high degree of selectivity and sensitivity. UHPLC-Q-TOF-MS allows the identification of capsaicinoids with minimal error based on exact experimental mass values. HRMS/MS data combined with the proposed identification strategy allows identification of novel or less studied capsaicinoid-derived compounds, being a very useful tool for metabolomic studies of this family of compounds, which would allow the improvement of knowledge about their toxicity or health impact.

## Supplementary Information

Below is the link to the electronic supplementary material.

Supplementary file1 (DOCX 814 KB)
