# Supplementary material for: Dispersive magnetic solid-phase extraction for capsaicinoid compounds in human serum using LC-HRMS: targeted and non-targeted approaches
Source: Anal Bioanal Chem. 2023 Jan 25;415(11):2133–45. doi: 10.1007/s00216-023-04544-7 (PMC10079705; doi:10.1007/s00216-023-04544-7)
Supplement: Supplementary file 1 — Supplementary file1 (DOCX 814 KB) [file 216_2023_4544_MOESM1_ESM.docx]

**Dispersive magnetic solid-phase extraction for capsaicinoid compounds in human serum using LC-HRMS: targeted and non-targeted approaches**

María Consolación Rodríguez-Palazón, Natalia Arroyo-Manzanares, Pilar Viñas*, Ignacio López-García, Manuel Hernández-Córdoba, Natalia Campillo

| **Table S1** UHPLC-Q-TOF-MS parameters of the studied compounds | | | | | |
| --- | --- | --- | --- | --- | --- |
| Compound | Molecular Formula | *m/z*  Theoretical | *m/z* Experimental | Error  (ppm) | Product ions (*m/z*) |
| CADB | C_16_H_23_NO_3_ | 278.1751 | 278.1764 | 2.8 | 151.0755 |
| PCAP | C_17_H_27_NO_3_ | 294.2064 | 294.2075 | 2.0 | 137.0602; 170.1505 |
| CAP | C_18_H_27_NO_3_ | 306.2064 | 306.2077 | 2.5 | 137.0602; 182.1545 |
| DCAP | C_18_H_29_NO_3_ | 308.2220 | 308.2224 | 1.2 | 137.0595; 184.1699 |

| **Table S2** Slopes^a^ of standard additions calibration graphs (L µg^-1^) | | | |
| --- | --- | --- | --- |
| Compound | Aqueous | Serum 1 | Serum 2 |
| PCAP | 0.0066±0.0001 | 0.0091±0.0001 | 0.0105±0.0006 |
| CAP | 0.0100±0.0002 | 0.0126±0.0001 | 0.0146±0.0009 |
| DCAP | 0.0091±0.0001 | 0.0122±0.0003 | 0.0129±0.0002 |
| ^a^ Mean value ± standard deviation (n=8) | | | |

| **Table S3** Structures, molecular formula and exact mass of described capsaicinoid-derived compounds | | |
| --- | --- | --- |
|  |  |  |
| CAP  C_18_H_27_NO_3_ *m/z* 305.19909 | DCAP  C_18_H_29_NO_3_ *m/z* 307.21475 | PCAP  C_17_H_27_NO_3_ *m/z* 293.19909 |
|  |  |  |
| Nordihydrocapsaicin  C_17_H_27_NO_3_ *m/z* 293.19909 | Homocapsaicin  C_19_H_29_NO_3_ *m/z* 319.21475 | Homodihydrocapsaicin  C_19_H_31_NO_3_ *m/z* 321.23040 |
|  |  |  |
| Nornorcapsaicin  C_16_H_23_NO_3_ *m/z* 277.16778 | Nornordihydrocapsaicin  C_16_H_25_NO_3_ *m/z* 279.18344 | M1  C_18_H_25_NO_3_ *m/z* 303.1834 |
|  |  |  |
| M2  C_18_H_27_NO_4_ *m/z* 321.1940 | M3  C_18_H_27_NO_4_ *m/z* 321.1940 | M4  C_18_H_25_NO_3_ *m/z* 303.1834 |
|  |  |  |
| M5  C_18_H_27_NO_4_ *m/z* 321.1940 | M6  C_17_H_25_NO_3_ *m/z* 291.1834 | M7  C_18_H_27_NO_4_ *m/z* 321.1940 |
|  |  |  |
| M8  C_18_H_27_NO_4_ *m/z* 321.1940 | M9  C_18_H_25_NO_3_ *m/z* 303.1834 | M10  C_17_H_23_NO_3_ *m/z* 289.1677 |
|  |  |  |
| M11  C_17_H_23_NO_3_ *m/z* 289.1677 | M12  C_17_H_25_NO_4_ *m/z* 307.1783 | M13  C_18_H_27_NO_5_ *m/z* 337.1889 |
|  |  |  |
| M14  C_18_H_27_NO_5_ *m/z* 337.1889 | M15  C_18_H_25_NO_4_ *m/z* 319.1783 | M16  C_18_H_25_NO_4_ *m/z* 319.1783 |
|  |  |  |
| M17  C_18_H_25_NO_4_ *m/z* 319.1783 | M18  C_18_H_25_NO_4_ *m/z* 319.1783 | M19  C_18_H_25_NO_4_ *m/z* 319.1783 |
|  |  |  |
| GSH11  C_27_H_40_N4O_10_S *m/z* 612.2465 | GSH12  C_28_H_42_N_4_O_11_S *m/z* 642.2570 | GSH13  C_38_H_57_N_7_O_16_S_2_ *m/z* 931.3297 |
|  |  |  |
| CAP-dimer  C_36_H_52_N_2_O_6_ *m/z* 608.3825 |  |  |

| **Table S4** Ion fragment 1 for the known capsaicinoid-derived compounds |
| --- |
|  |
| CAP, DCAP, PCAP, NDCAP, HCAP, HDCAP, NCAP, NDCAP, M1, M2, M3, M4, M15  C_8_H_9_O_2_^+^ *m/z* 137.0597 |
|  |
| M5, M7, M13, M14, M16, M17, M18, M19  C_8_H_9_O_3_^+^ *m/z* 153.0546 |
|  |
| M6, M10, M11, M12  C_7_H_7_O_2_^+^ *m/z* 123.0440 |
|  |
| M8  C_8_H_10_NO_3_^+^ *m/z* 168.06552 |
|  |
| M9  C_8_H_10_NO_2_^+^ *m/z* 152.0760 |


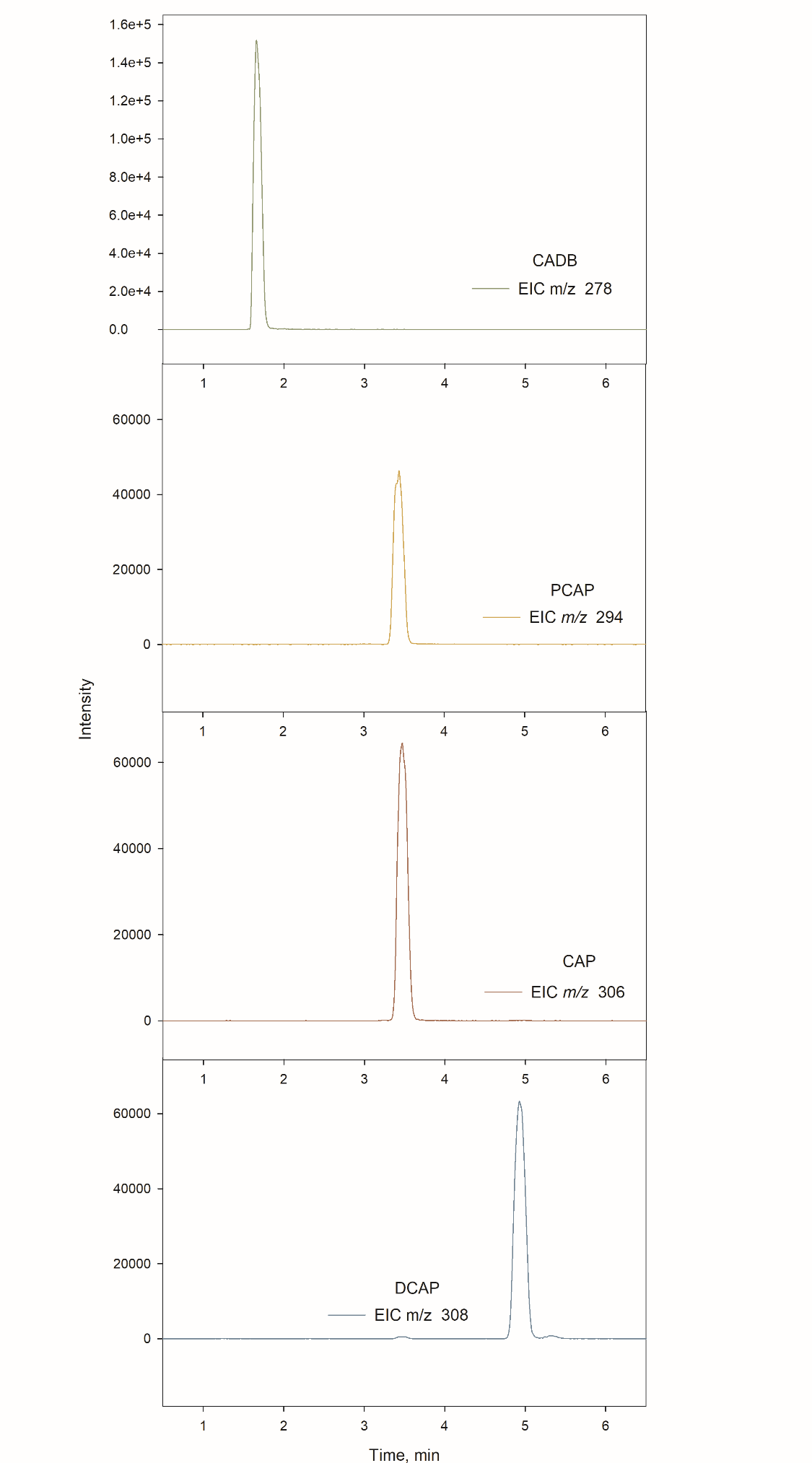


**Fig. S1** EICs for the capsaicinoids and the IS obtained by applying the optimized DMSPE with UHPLC-HRMS method for a serum sample fortified at 5 μg L^-1^.

**Fig. S2** HRMS/MS spectrum of DCAP standard
